# Marfanoid to Mortality: A Case Report on Sudden Cardiac Death Due to Aortic Dissection in a Young Male With Marfanoid Habitus

**DOI:** 10.7759/cureus.50651

**Published:** 2023-12-17

**Authors:** Saket Toshniwal, Anuj Chaturvedi, Sourya Acharya, Gajendra Agrawal, Sunil Kumar

**Affiliations:** 1 Medicine, Jawaharlal Nehru Medical College, Datta Meghe Institute of Higher Education and Research, Wardha, IND; 2 Cardiology, Jawaharlal Nehru Medical College, Datta Meghe Institute of Higher Education and Research, Wardha, IND

**Keywords:** marfan syndrome, fibrillin 1, cardiovascular complications, sudden cardiac death, aortic dissection, marfanoid habitus

## Abstract

This compelling case study unravels a tragic narrative of a 40-year-old male with Marfanoid habitus, navigating the intricate web of Marfan syndrome (MFS) and succumbing to the devastating complications of aortic dissection. The patient’s journey underscores the challenges in managing this rare connective tissue disorder, emphasizing the critical interplay between genetic predisposition and cardiovascular pathology. Moreover, the lack of immediate operative intervention due to the critical condition emphasizes the crucial need for timely diagnosis and intervention. The journey from genetic mutation to cardiovascular complications in MFS or related marfanoid habitus is complex and multifaceted. This case study aims to navigate this intricate path, emphasizing the need for a nuanced understanding of the underlying molecular and structural changes. Furthermore, it reinforces the critical role of ongoing cardiovascular monitoring and surgical interventions to prolong survival and enhance the quality of life for individuals grappling with the challenges posed by MFS or related habitus.

## Introduction

Marfan syndrome (MFS), a rare autosomal dominant disorder affecting approximately one in 5,000 individuals globally, is associated with mutations in the *fibrillin-1* (*FBN1*) protein gene, disrupting the delicate balance of the extracellular matrix [1]. This mutation gives rise to a spectrum of clinical manifestations, with cardiovascular complications, notably aortic root dilatation leading to dissection and rupture, being a common cause of sudden cardiac death [2]. Conversely, individuals with marfanoid habitus, displaying physical traits resembling MFS, may or may not have the syndrome itself, necessitating additional diagnostic assessments for *FBN1 *mutations [3]. While musculoskeletal features often characterize MFS or marfanoid habitus, it is the cardiovascular manifestations that significantly impact long-term prognosis. The heightened vulnerability of blood vessels, primarily composed of connective tissues, places a particular emphasis on cardiovascular implications [4]. The altered microfibril structure in MFS renders the aorta less distensible, increasing susceptibility to structural failures under considerable forces. Unlike individuals with a normal genotype, those with MFS experience structural failure rather than elastic distension, necessitating vigilant monitoring and surgical interventions to mitigate cardiovascular risks [5]. This case study delves into the narrative of an individual grappling with MFS-related marfanoid habitus, marked by Type III DeBakey aortic dissection and profound cardiovascular complications. The subsequent development of an aortic arch pseudoaneurysm, coupled with thrombus formation and sudden death, underscores the ongoing challenges in managing this syndrome. These complexities underscore the need for an aggressive surgical strategy, echoing the broader theme of clinical management in individuals with MFS or marfanoid habitus. This case study contributes to the evolving discourse on marfanoid habitus and its cardiovascular implications, offering insights for clinicians, researchers, and healthcare providers. By exploring the clinical course, diagnostics, and management decisions, we aim to enhance collective understanding and foster a proactive approach to addressing cardiovascular complexities in individuals with marfanoid habitus. This case study aims to increase the importance of evaluation in patients not fulfilling the criteria for MFS but having marfanoid habitus and to screen them thoroughly for cardiovascular structural defects to prevent terminal cardiac events as in our case [6].

## Case presentation

**Figure 1:**
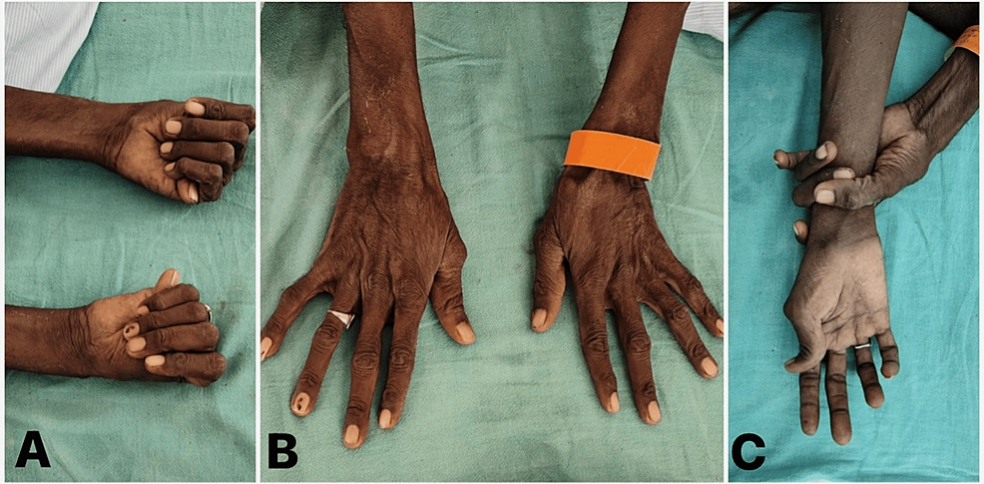
Clinical characteristics of marfanoid habitus. (A) Thumb extending well beyond the ulnar border of the hand when overlapped by fingers suggestive of a positive “thumb sign.” (B) Elongated spider-like fingers suggestive of “arachnodactyly.” (C) A positive “wrist sign” where the thumb overlaps the fifth finger on grasping the contralateral wrist.

A 40-year-old male with clinical characteristics of marfanoid body habitus, as shown in Figure 1, presented with an acute onset of severe chest pain radiating to the back, described as tearing in nature.

**Figure 2:**
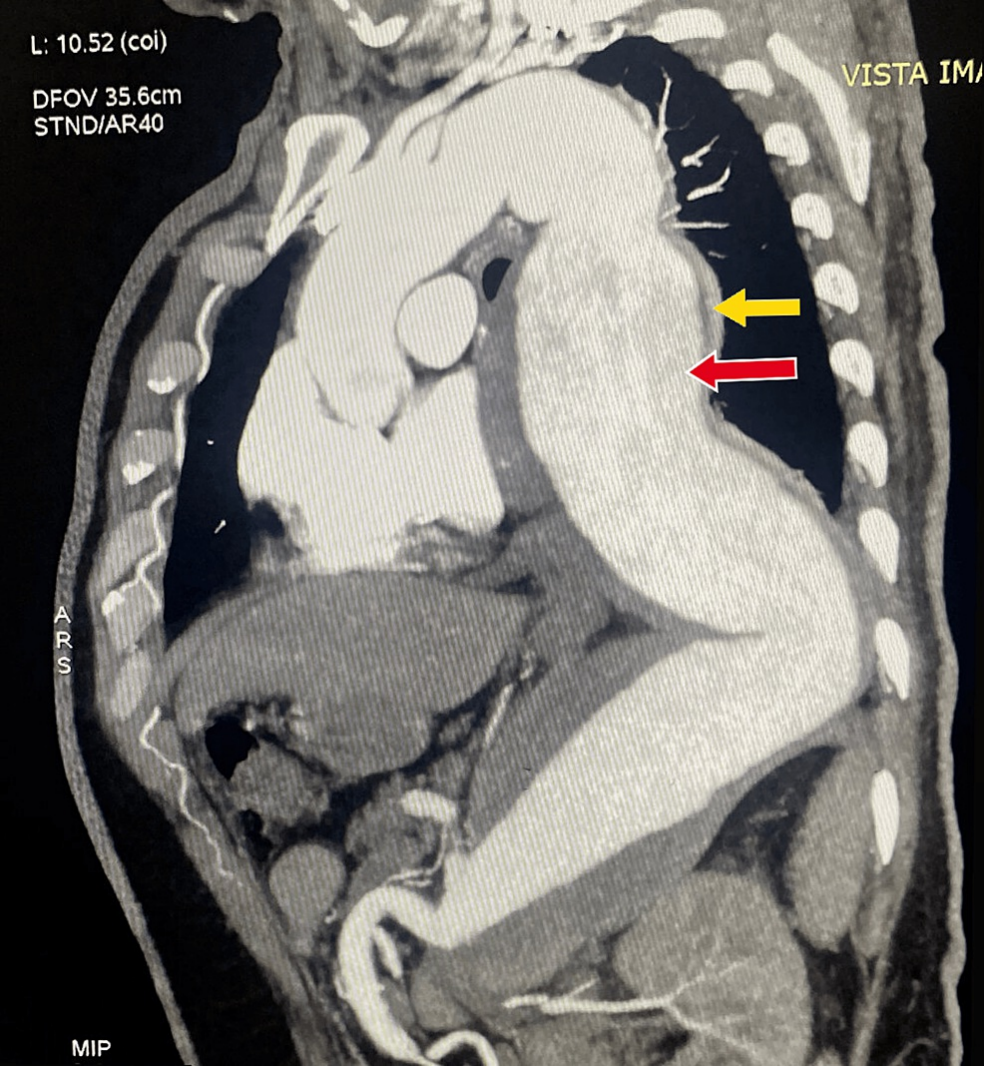
Type III DeBakey aortic dissection. Computed tomography aortogram sagittal section showing Type III DeBakey aortic dissection, originating at the T3 level in the thoracic aorta, distal to the left subclavian artery, showcasing a smaller true lumen (yellow arrow) and a larger false lumen (red arrow).

The patient’s blood pressure was recorded at 190/110 mmHg with wide pulse pressure, indicative of a hypertensive emergency with a widened mediastinum on chest X-ray, raising suspicions of thoracic aortic aneurysm and aortic dissection involving the ascending and proximal descending aorta. To further assess the extent of the condition, an urgent computed tomography (CT) aortogram was recommended. The imaging unveiled a Type III DeBakey aortic dissection, originating at the T3 level in the thoracic aorta, distal to the left subclavian artery, as shown in Figure 2. The dissecting flap extended inferiorly along the left posterolateral aspect of the abdominal aorta, showcasing a smaller true lumen and a larger false lumen. Notably, intramural hematoma within the false lumen exerted a mass effect on the left lower lung segment. While the celiac trunk displayed mild narrowing, other major arteries, including the celiac trunk, superior mesenteric, bilateral renal, and bilateral common iliac arteries, appeared unaffected.

Unfortunately, the patient rapidly progressed into sudden shock, precluding immediate surgical intervention. Regrettably, within the next five hours, the patient succumbed to the complications secondary to the aortic dissection followed by rupture of the aortic aneurysm leading to sudden shock. Underscoring the critical nature of aortic dissection in individuals with marfanoid habitus and emphasizing the imperative for swift diagnosis and intervention to improve outcomes in this high-risk cohort.

## Discussion

Marfanoid habitus, an autosomal dominant connective tissue disorder, resembles MFS and is characterized by a tall and slender physique, dolichostenomelia, arm span larger than height, arachnodactyly of hands and feet, little subcutaneous fat, and muscle hypotonia with a positive thumb and wrist sign, as observed in this case [7]. These also serve as a red flag clinical marker prompting further investigation due to the association between MFS and cardiovascular complications. It is often associated with mutations in the *FBN1* protein gene, unveiling a cascade of events leading to structural changes in the extracellular matrix, particularly in microfibrils, thereby predisposing individuals to a range of clinical manifestations, notably cardiovascular complications [2]. Aortic disease, given the role of *FBN1 *in providing structural support to the aortic walls through microfibrils. The altered microfibril structure in MFS renders the aorta less distensible, setting the stage for structural failures under the considerable forces experienced in this vital vessel. Unlike the elastic response seen in individuals with a normal genotype, the aorta in MFS exhibits structural failure under high stresses, setting the stage for a heightened propensity toward cardiovascular complications [1,3]. Unfortunately, the sudden death of the patient precluded the assessment of *FBN1 *mutations in this case.

The diagnostic journey in this case faced formidable challenges. The initial clinical presentation of tearing chest pain and a widened mediastinum on a plain chest X-ray triggered a swift diagnostic response, leading to a critical diagnostic examination, the CT aortogram. This imaging modality provided a detailed roadmap of the vascular intricacies, unveiling a Type III DeBakey aortic dissection. Notably, the imaging also revealed a complex scenario with a smaller true lumen, a larger false lumen, and an intramural hematoma. This imaging not only confirmed the severity of the aortic pathology but also revealed the intricacies of the vascular anatomy. The collapse and consolidation of the left lower lung segment due to mass effect added a nuanced layer of diagnostic complexity rarely seen in MFS cases, emphasizing the need for advanced imaging modalities and heightened clinical acumen in this case presenting with acute chest pain.

The vascular measurements revealed the extensive involvement of the descending abdominal aorta, unveiling the intricate vascular anatomy inherent to Marfan-associated aortic dissection [8]. The aortic arch pseudoaneurysm introduced an additional layer of complexities, which were further aggravated and complicated by thrombus formation. Moreover, the narrowing of the celiac trunk raised concerns about potential visceral perfusion compromise. These anatomical intricacies highlight the need for comprehensive assessment and ongoing monitoring in the management of Marfan syndrome, as complications can arise in various segments of the aorta, necessitating a tailored therapeutic approach.

The patient’s rapid progression to shock despite medical intervention highlights the aggressive nature of aortic dissection in the context of MFS. The compromised hemodynamics and the intricate vascular pathology in this case underscore the challenges faced by clinicians in stabilizing patients with such advanced cardiovascular complications [9]. The wide pulse pressure observed in the blood pressure readings further reflects the severity of the hemodynamic compromise and emphasizes the urgency for intervention [10]. The rapid clinical deterioration posed a considerable challenge, raising questions about the optimal timing and feasibility of operative intervention in such critical cases. The decision-making process in such scenarios involves a delicate balance between the urgency for surgical intervention and the patient’s overall stability [11].

This case prompts reflection on the limitations of current treatment modalities and the need for innovative therapeutic strategies tailored to the complexities of marfanoid habitus-associated aortic dissection. The complexities of this case emphasize the necessity of a multidisciplinary approach, involving cardiovascular surgeons, geneticists, and critical care specialists. Collaborative efforts are pivotal in navigating the intricate dynamics between genetic predisposition and cardiovascular pathology, especially in cases where surgical interventions may be challenging. As we move forward, research initiatives should focus on refining therapeutic approaches, enhancing risk stratification, and developing innovative surveillance strategies tailored to the intricate challenges posed by MFS or marfanoid habitus and associated aortic complications.

## Conclusions

In a nutshell, this case study not only illuminates the intricacies of managing aortic dissection in the context of marfanoid habitus but also highlights the pressing need for ongoing research and collaborative efforts to improve diagnostic precision, therapeutic interventions, and overall outcomes for individuals with the complexities of MFS. Aortic dissection in MFS is an important terminal event that needs to be taken into consideration; hence, awareness among practitioners for genetic evaluation of patients with marfanoid habitus and screening patients with marfanoid habitus not fulfilling the diagnostic criteria of MFS for cardiovascular structural defects are important to prevent mortality.
